# Comment on “Optimal centrifugal isolating of liposome–protein complexes from human plasma” by L. Digiacomo, F. Giulimondi, A. L. Capriotti, S. Piovesana, C. M. Montone, R. Z. Chiozzi, A. Laganá, M. Mahmoudi, D. Pozzi and G. Caracciolo, *Nanoscale Adv.*, 2021, **3**, 3824†

**DOI:** 10.1039/d2na00343k

**Published:** 2022-11-30

**Authors:** Rasmus Münter, Jens B. Simonsen

**Affiliations:** Department of Health Technology, Biotherapeutic Engineering and Drug Targeting, Technical University of Denmark (DTU) 2800 Kgs. Lyngby Denmark ramila@dtu.dk

## Abstract

In a recent paper in Nanoscale Advances, Digiacomo *et al.* conclude that centrifugation should be the method of choice for researchers who want to investigate the protein corona of liposomes for drug delivery in human plasma. In this Comment, we however propose the opposite – that centrifugation, in most cases, is unsuitable for isolating liposomes from human plasma. Our conclusion is based on the bulk literature on this and similar topics, and new experimental data based on formulations and protocols like the ones used by Digiacomo *et al.*

## Abstract 2

Centrifugation is unsuitable for isolating liposomes from human plasma, and should not be the method of choice for researchers wanting to investigate the protein corona of liposomes for drug delivery.

## Graphical abstract

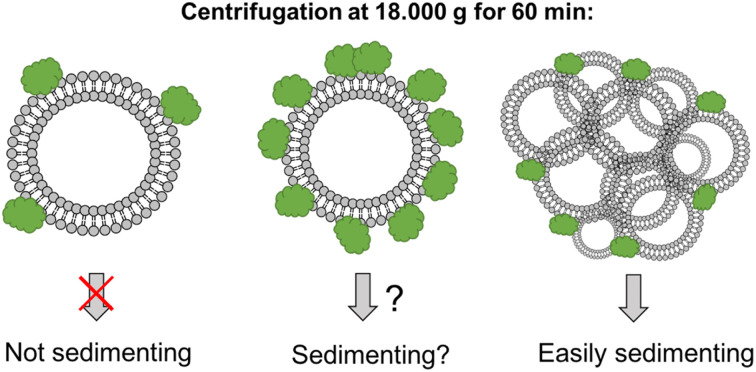

## 1. Introduction

The protein corona of drug delivery vehicles has in the past years received considerable interest. This layer of proteins, adsorbed onto the surface of nanoparticles when exposed to a biological environment such as blood plasma, are what cells effectively “see”. Hence, the protein corona is proposed to govern the interaction with cells in the body, both with respect to targeting and off-targeting effects within drug delivery. The protein corona is therefore, in many cases, decisive for the fate of the drug cargo in or on the nanoparticle.^1,2^

The protein corona field has started to recognize how challenging the study of the protein corona on nanomedicines is. A key challenge is related to the isolation of the drug delivery nanoparticles from biological fluids such as human plasma, which contain protein complexes and biological nanoparticles with similar physical (incl. size and density) and chemical (lipid-based surface) properties.^3^ These bionanoparticles and protein complexes may be co-isolated with the synthetic nanoparticles,^4,5^ and hence contaminate the isolates with proteins not actually associated to the nanocarriers, effectively leading to false conclusions on both quantitative and qualitative aspects of the protein corona.^3^ To overcome these hurdles, the methodologies used to study the protein corona are becoming still more sophisticated.^6–9^ A non-fulfilled goal is to establish efficient and well-aligned protocols for nanocarrier isolation, including proper controls, which make it possible to obtain high yield and purities of the nanocarrier isolate and to compare studies across laboratories.^2,10,11^ As part of this activity, researchers are beginning to compare the different available methodologies, to determine which is the optimal method for investigating the protein corona of various types of nanoparticles.^4,6,7^

In the recent paper,^12^ Optimal centrifugal isolating of liposome–protein complexes from human plasma, Digiacomo *et al.* state that “it is clear that centrifugation should be the technique of choice for studies on the liposome–protein corona”. Here, we challenge this statement by presenting findings from the bulk literature and providing new experimental data. This information all supports the notion that centrifugation, in most cases, is inadequate to isolate high yield and pure liposomes with and without a protein corona from human plasma. Altogether, we end up at the conclusion that researchers who want to study the protein corona of drug delivery liposomes, should refrain from solely using centrifugation. Instead, we suggest alternative more suitable methodologies.

## 2. Theoretical considerations on pelleting liposomes using centrifugation

Before discussing the data presented by Digiacomo *et al.* (“the authors”), we will briefly present the basic principles behind centrifugation, as the centrifugation technique is the focal point of this comment. The theoretical consideration will allow the reader to understand which parameters that could affect the sedimentation rate of a nanoparticle in different media. The sedimentation rate of a spherical particle (*ν*) can be described by Stokes' law:
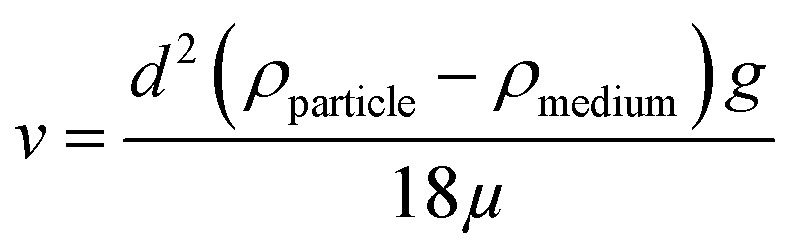


The particle diameter corresponds to *d*, *ρ*_particle_ is the particle density, *ρ*_medium_ is the density of the medium, *g* is the gravitational force and *μ* is the viscosity of the medium. From this equation, it is clear that the sedimentation rate of a spherical particle depends on its diameter and on the density of the particle in relation to the density of the surrounding medium.

As liposomes consist mainly of water, surrounded by a thin lipid bilayer shell, we would intuitively expect them to be quite challenging to pellet, because their density will be very similar to the density of the surrounding medium (*i.e.* water or saline). Indeed, centrifugations above 100 000*g* for 20–150 minutes have previously been used to pellet multilamellar, brominated, or aggregated liposomes, while leaving unilamellar, non-brominated and non-aggregating liposomes in solution.^13,14^ With this in mind, we would expect that higher centrifugation speeds and longer centrifugation times than the 21 400*g* for 15–60 minutes used by Digiacomo *et al.*, would be required in order to pellet individual liposomes containing no or a small amount of adsorbed proteins. However, the centrifugation parameters required for sedimentation of liposomes in plasma are not straightforward to assess. First, plasma has higher density^15^ and viscosity^16^ than water and PBS, which would reduce the sedimentation rate of the liposomes compared the rate in water or PBS, calling for longer centrifugation and/or higher centrifugation forces. Second, creation of a protein corona on the surface of a liposome, would proposedly increase both the density and the size of the particle, reducing the required centrifugation force and time. Predicting these counter-acting factors is not straightforward, and instead the efficiency of the methodology is best assessed experimentally. Keeping the parameters controlling sedimentation rate in mind, we therefore continued by experimentally investigating the ability of the centrifugation protocol to pellet liposomes with and without a protein corona.

## 3. Pelleting cationic liposomes

Proceeding from the theoretical considerations above, we next investigated experimentally if the centrifugation procedure used by Digiacomo *et al.* was able to pellet liposomes.

**Fig. 1:**
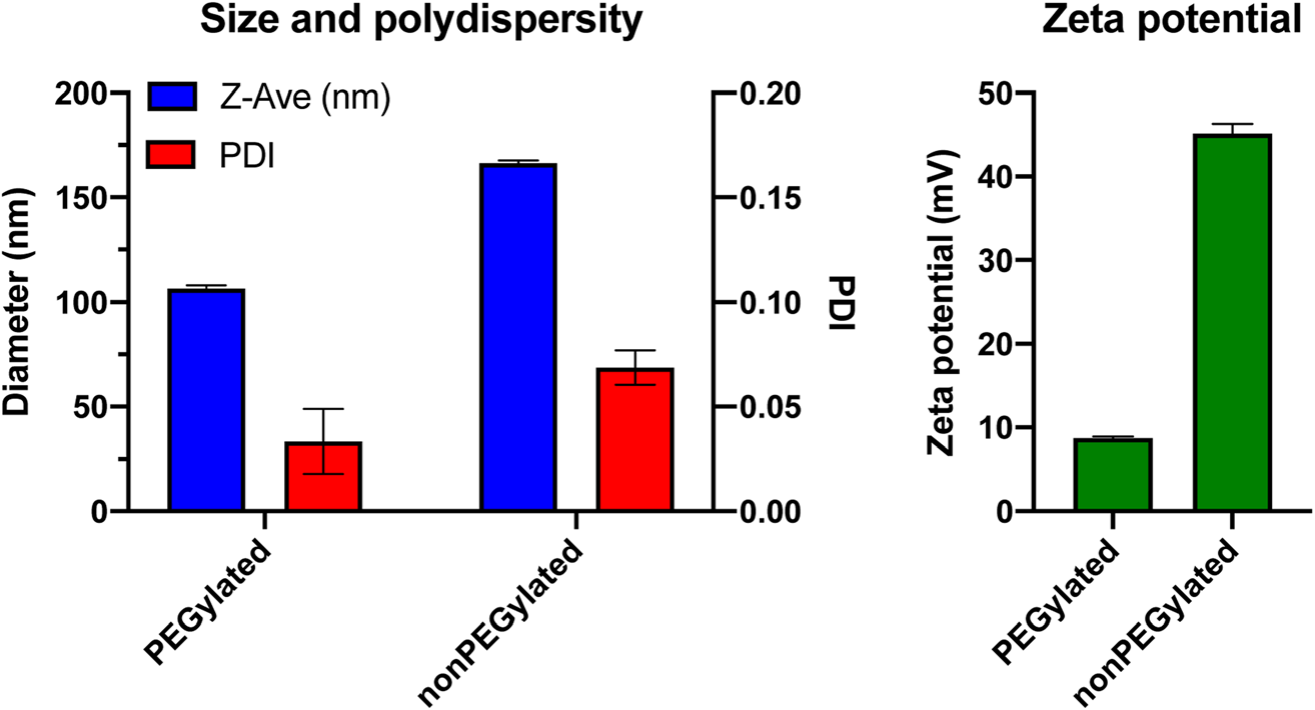
Physicochemical characteristics of the PEGylated and nonPEGylated cationic liposomes used in this Comment. Size (*Z*-ave) is measured by dynamic light scattering (DLS) in PBS, zeta potential is measured at pH 7.4 in a buffer containing 10 mM HEPES and 5% glucose. Results shown are the mean of three individual measurements on the same batch of liposomes, error bars represent the standard deviation.

To put us in a proper position to evaluate the studies by the Digiacomo *et al.*, we prepared liposomes similar to those used by the authors: a non-PEGylated liposome formulation (50 mol% DOTAP and 50 mol% DOPE), and a PEGylated version consisting of DOTAP : DOPE : DOPE-PEG in a molar ratio of 50 : 35 : 15. We added 0.1 mol% of the fluorescent lipid DiI to these lipid mixtures for liposome tracking. We chose DiI (containing two saturated 18 carbon atoms (C18) alkyl chains), rather than the DHPE-anchored probe used by the Digiacomo *et al.*, as we have previously demonstrated that fluorescently labeled lipids with C16 alkyl/acyl chain anchors (such as DPPE/DHPE), have a higher propensity to dissociate from liposomes in blood plasma than those with C18 alkyl/acyl chains, and hence may not actually trace solely the liposomes but also other plasma components.^17^ Briefly, lipids were dissolved in *tert*-butanol : water 9 : 1 and mixed to the desired ratio followed by overnight lyophilization to remove the solvent. After rehydration of the lipid film in PBS and 1 h magnetic stirring at 45 °C, the lipid suspensions were exposed to 11 freeze/thaw cycles to reduce multilamellarity, by repeatedly dipping the vials in liquid nitrogen and a 65 °C water bath. Then, the liposomes were extruded 21 times through a polycarbonate filter with 100 nm pores, using an Avanti Mini Extrusion kit. The final lipid concentration was 5 mM total lipid for both formulations. To determine the size, polydispersity and zeta potential of the liposomes, they were characterized by dynamic light scattering (DLS) using a Malvern Zetasizer ZS (Fig. 1). The PEGylated liposomes had a mean size of 106.6 ± 1.6 nm with a polydispersity index of 0.03. The nonPEGylated liposomes were larger with a mean size of 166.4 ± 1.3 nm and a PDI of 0.07. The zeta potential in 10 mM HEPES (pH 7.4) was +8.7 mV for the PEGylated formulation, whereas the nonPEGylated liposomes – where no PEG layer was shielding the charge – were highly cationic with a zeta potential of +45.2 mV (Fig. 1). As the liposomes used by Digiacomo *et al.* had the exact same lipid compositions (except for the choice of fluorophore-labeled lipid), we assume that their liposomes had similar physicochemical characteristics. These properties were, however, not reported in their paper.^12^

In order to investigate quantitative aspects of the protein corona of a nanoparticle, the methodology employed should be able to address both if a rich protein corona is formed on a nanoparticle, but also address the case where no protein corona is formed. To test the ability of the centrifugation procedure to pellet liposomes without adsorbed proteins, we diluted the liposomes to a final concentration of 1 mM lipid in PBS, and incubated the sample at 37 °C for 60 minutes. We then exposed the samples to a 60 minute centrifugation at 18 000*g* and 4 °C, which is the longest centrifugation time employed by Digiacomo *et al.*, but a slightly lower centrifugation force (see discussion below). Before and after this centrifugation step, 20 μL was taken out of the supernatant for quantifying the amount of liposomes in the sample, by measuring the fluorescence intensity from the DiI label. To avoid that environmental differences in the samples gave rise to different optical properties of the fluorophores, the 20 μL sample was diluted 1 : 10 in EtOH before measuring fluorescence at 550/570 ex/em using a microplate reader.

**Fig. 2:**
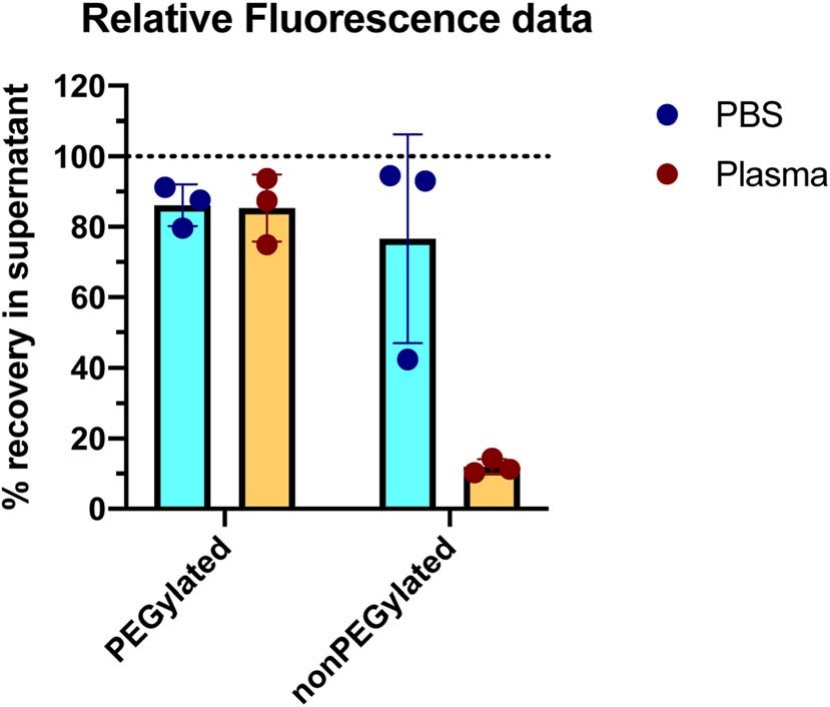
Recovery of liposomes in the supernatant after centrifugation for 60 minutes at 18 000*g*. Results are shown for both PEGylated and nonPEGylated liposomes consisting of DOTAP, DOPE and DOPE-PEG. The experiment was carried out in both the absence (PBS) and presence (plasma) of proteins from human blood plasma. The percentages are based on the DiI fluorescence measured in the supernatant after centrifugation, compared to the DiI fluorescence of the sample before centrifugation. *N* = 3. Error bars represent the standard deviation.

**Fig. 3:**
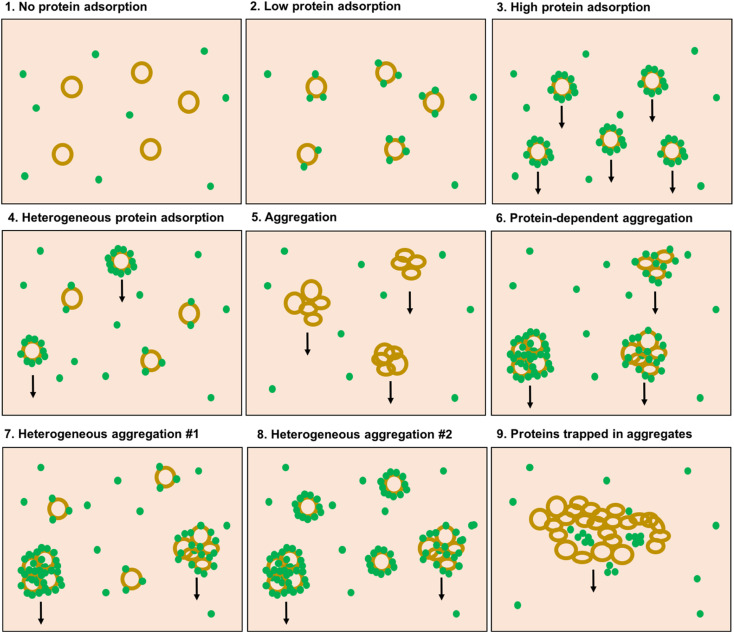
Possible scenarios for protein adsorption to liposomes in plasma, and aggregation of liposomes in plasma and the proposed likelihood to be sedimentated at the centrifugation condition used by the Digiacomo *et al.* (illustrated by arrows). (1) No proteins adsorb to the liposomes, (2) low adsorption of proteins to the liposomes, (3) high adsorption of proteins to individual liposomes. (4) Heterogeneous protein adsorption to the liposomes: some liposomes adsorb more protein than others. (5) Liposome aggregation without the involvement of plasma proteins. (6) Aggregation of liposomes mediated by proteins. (7) Heterogeneous aggregation: some liposomes have high protein adsorption and form aggregates, other liposomes only adsorb low number of protein. (8) Heterogeneous aggregation: some liposomes form aggregates with high protein binding, other liposomes adsorb a high number of proteins but do still not aggregate. (9) Proteins and bionanoparticles are trapped in large liposome aggregates and are co-pelleted with the aggregates.

The outcome of the experiment is illustrated in Fig. 2, showing the DiI fluorescence (representing the liposomes) in the supernatant after centrifugation, relative to the fluorescence intensity in a sample taken before the centrifugation step (the same analysis method as used by Digiacomo *et al.*). An average of 86% of the PEGylated liposomes were remaining in the supernatant after the centrifugation step. Those liposomes that do pellet, could be a subpopulation of multilamellar or agglomerated liposomes. For the nonPEGylated liposomes, an average of 76% of the liposomes were remaining in the supernatant after centrifugation. With only one out of six samples as an exception, both PEGylated and nonPEGylated liposomes in PBS could in general not be pelleted by centrifuging 18 000*g* for 60 min (>80% in the supernatant). In plasma, which has higher viscosity and density than PBS,^15,16^ would the sedimentation rate be even lower. Hence, in such a medium, the sedimentation propensity of the liposomes would be even lower than in PBS (at least, in a situation where no proteins adsorbed to the liposome and no liposome-aggregates are formed). Hence, the centrifugation methodology is not able to demonstrate the negative outcome of a protein corona experiment, which is the outcome: “no proteins adsorb to the liposomes” (scenario #1 in Fig. 3).

We next investigated if the liposomes could be pelleted after incubation in human plasma. We therefore incubated the liposomes in 80% freshly acquired human EDTA plasma (attempting to address the protein corona formed under conditions as close to an *in vivo* situation as possible) for 60 minutes at 37 °C: this corresponds to the highest of three plasma concentrations used by Digiacomo *et al.* and the same incubation time and temperature. In this experiment, the final concentration of liposomes was also 1 mM lipid (the concentration used by Digiacomo *et al.* was not reported). This corresponds roughly to the expected liposome concentration in human blood following infusion of PEGylated liposomes in the clinic.^3^ The same centrifugation protocol and fluorescence measurements were carried out as described above for the samples with liposomes in PBS. The results are shown in Fig. 2. For PEGylated liposomes, the recovery of liposomes in the supernatant was similar as what we observed in PBS: 85% of the liposomes were found in the supernatant, hence only a minority (15%) of liposomes was pelleted. This would even be the maximum expected fraction of recovered pelleted liposomes in a protein corona experiment, as only one centrifugation step without any washing steps was performed, in contrast to Digiacomo *et al.* who washed their pellet three times in PBS. In general, PEGylated liposomes can neither be completely pelleted in the absence or presence of plasma proteins under the conditions we and Digiacomo *et al.* have used. For the nonPEGylated liposomes, however, only 12% of the liposomes were recovered in the supernatant after the centrifugation, which represents a huge decrease compared to the 76% in PBS. For this formulation, the presence of the plasma proteins resulted in most of the liposomes to be pelleted.

Arguably, the liposomes thus become pelletable when a protein corona is formed on them: bound proteins would increase the density and size of the particle, and hence increase the sedimentation rate of the particle according to Stokes' law. Both our results, and the results by Digiacomo *et al.*, would in that case indicate that PEGylated liposomes have so low protein adsorption that they cannot be pelleted (scenario #2 in Fig. 3), whereas nonPEGylated liposomes have so high protein binding that they can be pelleted (scenario #3 in Fig. 3). This explanation is however problematic, as it turns the existence of a rich protein corona into a self-fulfilling prophecy: if a protein corona is formed, then we are able to measure it. If a protein corona is not formed, or if it is very sparse, we can however not observe that this is the case, as the liposomes we want to investigate are not isolated for further analysis.

Importantly, there was no noteworthy difference between the amount of cationic PEGylated liposomes being pelleted in the absence or presence of plasma proteins (Fig. 2): hence, the presence of the plasma proteins does not change the density and size of these liposomes, or is counter-balanced by the increase in intensity and viscosity of the medium. The most meaningful interpretation to this similarity between the two samples, is that very few proteins are adsorbing to the cationic PEGylated liposomes (and potentially no proteins at all). This would mean that the pelleted proteins measured by Digiacomo *et al.*, co-isolated with their cationic PEGylated liposomes according to Fig. 4–6 of their paper, could, in principle, represent something else than a protein corona, such as protein aggregates or bionanoparticles. It should be mentioned though, that very little protein was observed in their liposome-free (control) plasma sample. That said, we cannot rule out that the presence of liposomes in the plasma sample can induce protein aggregation,^18,19^ without these aggregates binding to the liposomes.

Our results in plasma mirror the results presented by Digiacomo *et al.*: they do also find that approx. 80% of the nonPEGylated liposomes are pelleted after 60 minutes of centrifugation, whereas only 10–20% of the PEGylated liposomes are pelleted after similar conditions (Fig. 4a in the article). When only 10–20% of the PEGylated liposomes can be pelleted, it can be questioned whether the measured protein corona on this subset of liposomes then really be said to be representative for the protein corona on the total liposome population in the sample. When only a minority of the liposomes is pelleted, one could argue that this is the subpopulation of liposomes on which a protein corona is formed (or close to the bottom of the tube at the beginning of the centrifugation), whereas those liposomes that stay in the supernatant are the subpopulation of liposomes with little or no protein adsorbed (scenario #4 in Fig. 3). In principle, the assay could be used to measure the protein corona on the subpopulation of liposomes that pelleted. However, we will not be able to conclude anything about the quantitative aspects of protein corona (typically reported in terms of protein binding values *P*_B_ calculated as gram protein per mole lipid^20–22^) on non-pelleted liposomes. The non-pelleted liposomes could both have a dense corona formed on them (but not enough to make them pelletable), or no proteins adsorbed at all. When analyzing only a subpopulation of the liposomes, we risk making false conclusions about the general protein adsorption tendencies of the liposome formulation we are studying.

On a side note, it is becoming still more evident, that protein adsorption to nanoparticles is a heterogeneous phenomenon,^23–25^ which may be due to underlying heterogeneity (in terms of size, uni- and multilamellarity, structure, surface charge, and lipid composition) of the liposomes being studied.^26^ The non-pelleted liposome population may therefore contain important and valuable information that is lost when the centrifugation methodology does not allow to study it.

In conclusion, the centrifugation assay can only in some instances be used to isolate cationic liposomes from plasma. The procedure is therefore not generally suitable for studying the protein corona of liposomes.

## 4. Pelleting liposomes with low protein adsorption

Digiacomo *et al.* claim that cationic nonPEGylated liposomes are used, because these are “a gold-standard for gene and drug delivery applications”.^12^ While cationic liposomes are applicable for *in vitro* transfection (such as lipofectamine), we are however not familiar with any clinically approved cationic liposomes. Cationic particles tend to agglomerate in plasma and either get quickly cleared,^27,28^ or get stuck in the lungs due to the narrow lung capillaries^29,30^ and may potentially also induce toxic responses.^27,31,32^ Hence, the approved gene therapy (Onpattro) and the mRNA-based vaccines (Spikevax and Comirnaty) rely on ionizable lipids, which carry a neutral charge in physiological pH, and become cationic in an acidic environment (such as upon endocytosis).^33,34^ The protein corona on weakly anionic or a close-to neutrally charged lipid nanoparticles may therefore be more interesting to most researchers, because they are more clinically relevant.^35,36^

Several papers in the literature have attempted to use centrifugation based approaches to investigate the protein corona of clinically approved (non-cationic) PEGylated liposome formulations such as the stealth formulations traded under the names Doxil, Caelyx and LipoDox. For example, Kristensen *et al.* found that an average of 80% of stealth liposomes remained in the plasma supernatant after centrifuging 17 900*g* for 30 minutes.^4^ Similarly, a recent study by Hacene *et al.* found that 98% of the liposomes remained in the plasma supernatant after centrifuging 15 000*g* for 120 minutes.^7^ Another recent study by Pattipeiluhu *et al.* found that 90% of the clinically used liposome formulations AmbiSome and Myocet (non-PEGylated liposome products) remained in the plasma after a centrifugation step of 17 500g for 15 min.^8^ Pattipeiluhu *et al.* thereafter performed three washing steps with PBS: a procedure commonly used in the literature employing centrifugation for isolation of liposomes from plasma.^37–44^ After these washing steps, Pattipeiluhu *et al.* found almost no liposomes in the pellet.^8^ Similar results were obtained by Chu *et al.*, who recovered less than 1% of their stealth liposomes after centrifuging 14 000*g* for 30 minutes with three washing steps in PBS.^6^ In the latter study, the method was compared to liposomes isolated using affinity chromatography, finding that the protein/lipid ratio obtained by centrifugation was 4.3-fold lower than using chromatography, clearly highlighting a loss of valuable information when using centrifugation. Taken together, we are not the first to report that centrifugation is unsuitable for isolation of liposomes from plasma.

The ability to study (the most likely very sparse^45^) protein binding to stealth formulations is however of immense importance, as such formulations are prone to side effects such as accelerated blood clearance phenomenon and complement-mediated pseudo-allergies.^46–50^ Even for formulations with very sparse protein adsorption, the few proteins that do bind may hence have detrimental effects on their *in vivo* performance. Hence, it is of utmost importance for an assay used to study protein corona, that it is also useful for studying liposomes with low protein binding.

In conclusion, several studies in the literature support that the centrifugation methodology is not able to isolate clinically relevant liposome formulations from plasma. The method is therefore not suitable for investigating the protein corona of currently clinically relevant liposomes.

## 5. Liposomes that do pellet: rich corona or aggregation?

In the centrifugation study above, we concluded that liposomes with no or low protein binding cannot be pelleted efficiently by the centrifugation protocol used by us and Digiacomo *et al.*^12^ We did however observe that 88% or the nonPEGylated cationic liposomes were able to pellet after incubation in plasma. In absence of plasma, the same ability to be pelleted was not seen for the nonPEGylated formulation. Based on the discussion above, an obvious explanation for this change in sedimentation propensity could be that a rich protein corona is formed on the particles that do pellet. However, as we shall discuss below, there could also be other explanations.

Given the popularity of the centrifugation method in literature for the isolation of liposomes with a protein corona, the method would at a first glance seem to be well-validated. When centrifugation is used to conclude that a rich protein corona is formed on liposomes, it is in some cases supported by standard characterization methodologies. Going through the literature, we learned that DLS is a technique that is commonly used to verify the presence of protein corona on liposomes and thus support findings from centrifugation assays.^37,40–44^ Specifically, the size of the liposomes in PBS is compared to the size of the liposomes in plasma. At a first glance, this makes sense: if the liposomes are larger in plasma than in buffer, the increase in size should be because a layer of proteins is formed on the particle surface, increasing its effective hydrodynamic diameter. On DOTAP:DOPE liposomes, it has for example been found that the particles increase in size from 140 to 220 nm in plasma,^44^ which would lead to the conclusion that a 40 nm thick layer of proteins is formed on the particle, causing an increase in diameter of 80 nm.

However, we need to appreciate, that plasma contains many types of biological nanoparticles,^3^ that also consist of lipids and proteins. These bionanoparticles include various types of lipoproteins with sizes ranging from 5 to 1000 nm^3^, and extracellular vesicles with a size of 30–1000 nm^3^. If drug delivery liposomes are added to plasma in clinically relevant doses the number of drug delivery liposomes would be outnumbered manifold by these bionanoparticles.^3^ Taken this huge heterogeneity of particles in terms of size and type of particles into account, the DLS method falls short: whereas DLS is a strong technique for measuring the size of monodisperse nanoparticle formulations, it is challenged when more than one size population of particles are present. A common rule-of-thumb states that it is not possible to distinguish particles if the size difference is below a factor of three.^51,52^ To access the size distribution of liposomes in a complex solution, single-particle methods such as fluorescence correlation spectroscopy (FCS)^53,54^ or nanoparticle tracking analysis (NTA),^55^ in which the synthetic nanoparticles can be traced using fluorescent labels, are likely more applicable. DLS-based results, however, should be interpreted with care and can most likely not be trusted for samples of liposomes in plasma.

Had the particles first been separated from the bulk plasma by centrifugation, and the size of the corona-coated particles reconstituted in PBS then studied by DLS, we would encounter a similar problem as when quantifying the amount of protein per particle: only the liposomes with the highest number of proteins would be separated and included in the study, whereas the liposomes with low protein binding (and hence little or no increase in diameter) would not be included in the analysis. Further, the presence of bionanoparticles in the liposome–protein corona pellet will also challenge the downstream analysis.

Rather than being due to a rich protein corona being formed on the liposomes, an alternative explanation to why the nonPEGylated liposomes pellet, is aggregation. As mentioned above, one of the reasons that cationic liposomes are typically not used for intravenous injections, is due to their tendency to aggregate in human plasma and other biological environments.^27,30,56^ As can be seen from Stokes' law, the sedimentation speed does not just depend on the relative particle density, but also to the square of the particle size. Hence, large liposome aggregates are much easier to sediment than individual liposomes. A likely explanation for the ability of the nonPEGylated cationic DOTAP liposomes to quickly pellet at 18 000*g*, is that this formulation is not stable in plasma.

Studying aggregation of nanoparticles is however not straightforward. One way to do so is by studying the turbidity of the sample, but this will not allow for studying if the liposomes are part of the aggregates. Flow cytometry, however, offers a solution to this problem, and we recently demonstrated how that technique can be used to probe aggregation of cationic liposomes in blood plasma.^28^ Conventional flow cytometers are typically optimized for detecting large cells, whereas liposomes, proteins and bionanoparticles are difficult to detect due to their small size. However, aggregated liposomes are large enough to be detected even on a conventional flow cytometer. We recently demonstrated how both samples with liposomes alone as well as samples with liposome-free plasma resulted in very few detected events.^28^ In contrast, when nonPEGylated cationic liposomes (with less positive zeta potential than those we used in Fig. 1 and 2 of this comment) were added to the samples, a huge increase of events with scattering properties similar to leukocytes were detected by the cytometer.^28^ Comparable results were obtained when we performed the experiment with similar liposomes as those employed by Digiacomo *et al.* and in Fig. 2 of this Comment (see ESI Fig. S1†). The presence of the fluorescent liposome label in the detected events validated that they contained the liposomes. Such large liposome aggregates would most likely be very easy to pellet, even using low-speed centrifugation. Based on that study,^28^ we suggest that even liposomes with a less positive zeta potential than those used by Digiacomo *et al.*, can aggregate in plasma. We can therefore add a couple of more potential scenarios to Fig. 3: the liposomes that do pellet tend to aggregate, and these may then either be a significant proportion (scenario #6) or a minor subpopulation (scenario #7 and #8).

Depending on the experimental conditions, Digiacomo *et al.* were able to optimize the recovery, leading the authors to propose the optimal experimental conditions for investigating the protein corona. Based on our results, however, the actual effect leading to higher recovery using their centrifugation-based isolation method, is likely the tendency of the liposomes to aggregate in the different plasma concentrations. Digiacomo *et al.* indeed claimed that their liposomes had a tendency to aggregate when they employed a low plasma concentration of 5%, but did not discuss this possibility for the 80% plasma concentration. Here, we however propose that a significant proportion of the liposomes aggregate for the 80% plasma concentration too.

Aggregation as such may not be considered an issue for the researcher interested in the protein corona: the interesting question is if proteins adsorb to the lipid membranes. In principle, it does not matter if the liposomes stay as discrete individual particles, or as large agglomerates, even though we would need to abandon our idea of individual nanosized particles circulating in the blood. A consequence of the aggregation is furthermore that the increase in size would probably affect the circulation properties and cellular interactions much more than the effect from the proteins adsorbed. Hence, from a clinical perspective, aggregation should not be ignored. Another concern about the studies of the protein corona of aggregated liposomes is that the formation of liposome aggregates can trap plasma components as well, and thus pellet plasma proteins that are not directly associated with the liposomes (scenario #9 in Fig. 3).

We cannot for certain conclude, neither from our data nor the data presented by Digiacomo *et al.*, (or for that sake from any data in literature we are familiar with) whether the ability for cationic liposomes to sediment in plasma is because they aggregate or because they are covered by a rich protein corona. Based on our other works, we do find the aggregation scenario to be more realistic, but the various scenarious in Fig. 3, from scenario #3 through #8, are impossible to discriminate between with centrifugation alone, and need additional experiments to validate them. Furthermore, for the liposomes that do stay in the supernatant, we can not know for sure if they are sparsely coated with proteins (scenario #7 in Fig. 3), or densely coated with proteins (scenario #8 in Fig. 3): we will not be able to determine this, without a thorough study on the number of proteins per particle required for a particle to become pelletable. Herein lies the entire problem: using centrifugation, we have no idea which system we are actually studying. When the recovery of liposomes is only 15%, these are not representative for the overall ensemble, and we have no clue how the remaining 85% of the population behave. Hence, it is questionable if any unambiguous conclusions can be drawn from such an experiment.

In conclusion, the reason some cationic liposomal formulation can be isolated from plasma using the centrifugation methodology may be that these formulations aggregate in plasma, and not necessarily because the individual liposomes have high protein adsorption. This leads us to our overall conclusion for this Comment, which is in stark contrast to the statements by Digiacomo *et al.*: centrifugation should not be the method of choice for researchers who want to investigate the protein corona of liposomes for drug delivery.

## 6. Outlook towards improved separation methods

The aim of this comment is to highlight that unambiguous conclusions about the protein corona on the entire liposome population is, in many cases, not possible when using the centrifugation protocol proposed by Digiacomo *et al.* Further, Fig. 3 illustrates the many possible outcomes of liposome–protein interactions and lack of such that needs be considered when drawing conclusions based of liposomes isolated from centrifugation. Only in the event where the vast majority of the liposomes are pulled down during the centrifugation, a conclusion about the whole liposome population may be possible. However, even when this requirement is fulfilled, concerns about the formation of liposome aggregates leading to co-sedimentation of endogenous particles and proteins remain. Further, even in the case where the liposomes do not aggregate, co-isolation of endogenous bionanoparticles is a concern that always needs to be addressed by running a blank control.^3,4^ Finally we will present some suggestions on how the isolation techniques used in the protein corona field could be improved.

As briefly mentioned above, we did make some small amendments to the protocol used by Digiacomo *et al.* First, we exchanged the fluorescent label, to limit the risk of experimental errors associated with label dissociating into other plasma components.^17^ This change did however not seem to change the overall results, thus strengthening our conclusion that the lack of liposome sedimentation is truly because the liposomes do not pellet (and not because the tracer dissociates and does not co-pellet with the liposome). Second, we have performed our study at a slightly lower centrifugation speed (18 000*g*) than that used by Digiacomo *et al.* (21 400*g*). As the authors demonstrated in the ESI† of their work, that reducing the centrifugation speed to 13 700*g* only decreased the lipid recovery by 20%, we do not find that our adjustment have major impact on the overall conclusion. The reason for reducing the centrifugation speed relies on our choice of microcentrifuge tubes in which the experiment is performed, namely Protein LoBind tubes, which do not tolerate centrifugation speeds higher than 18 000*g* according to the specifications. Choosing appropriate tubes is important, as proteins and peptides tend to stick to regular microcentrifuge tubes,^57^ increasing the risk of carry-over. Indeed, carry-over is a concern for a centrifugation based setup, as removing all of the supernatant from the tube without disturbing the pellet is technically difficult. Even if all liquid is carefully removed, protein and lipid sticking to the sides of the vial could result in undesired carry-over (this matter is investigated in ESI Fig. S2†). Careful thought should therefore be given to the materials (tubes and pipette tips) used for such experiments.

When looking at ways to improve the centrifugation protocol, for example to allow PEGylated liposomes to be pelleted, an easy solution would at a first glance be to increase the centrifugation speed. For example, liposomes may pellet if centrifuged at >100 000*g* for >1 h.^14^ An issue with this is however, that it would also increase the likelihood of co-pelleting other plasma components such as lipoproteins, protein aggregates and extracellular vesicles.^3^ In principle, bionanoparticles could be removed from the plasma before incubation with liposomes (using *e.g.* ultracentrifugation or filtration), but valuable information regarding protein transfer from lipoproteins to liposomes would be lost. If increasing the centrifugation speed to 100 000*g*, the risk of compromising the integrity of the liposomes is also increased: Chu *et al.* demonstrated that doxorubicin tended to leak out of liposomes after 30 minutes of centrifugation at just 14 000*g*, clearly indicating that the liposome structures were disrupted even at relatively low speeds.^6^

Currently, no golden standard exists for an experimental setup for studying the protein corona of liposomes. Alternative popular methodologies include size-based separation methods such as Size Exclusion Chromatography (SEC)^4,45,58,59^ and Asymmetric Flow Field Flow Fractionation (AF4).^5,60^ As we have previously demonstrated,^4,45^ SEC also have some clear drawbacks, and one should thus be very careful with the control experiments when using SEC for studying the protein corona. Similar strict requirements to controls have been demonstrated for AF4.^5^ Specific immunoprecipitation methods for isolating liposomes and their protein corona have recently been demonstrated.^6,7^ While these methods are indeed very neat and specific, they may not capture the entire liposome population, and are typically limited to PEGylated liposomes rather than being generic. Pattipeiluhu *et al.* presented an elegant method relying on click chemistry, but even though it can be carried out with nonPEGylated nanocarriers, it still relies on specific labels.^8^ Magnetic separation of liposomes, challenged by the requirement for encapsulation of magnetic particles, has also been investigated.^61^ While being far from perfect, the size-based separation techniques (SEC and AF4) are currently the most effective tool in the toolbox of the protein corona researcher, in particular due to their high recovery of liposomes with both low and high protein adsorption.

While we may seem to have a negative view on the usefulness of the centrifugation methodology, we would however like to point out that we do not at all find it useless. Lipid aggregates that are easily pelleted could be used to capture and concentrate rare proteins, that are not easily detectable in pure plasma due to their relatively low abundance. Hence, the centrifugation protocol with unstable cationic liposomes could find its niche as a convenient diagnostic tool.^62–64^ Tang *et al.* recently demonstrated how protein-induced aggregation of liposomes could also be used to study drug leakage from liposomes using centrifugation.^65^ We however find the procedure unsuitable for studying the protein corona of non-aggregating nanoparticles, which from a clinical perspective are of much higher relevance than aggregating nanoparticles. We would furthermore like to stress that we do not find that centrifugation should be avoided when studying the protein corona of high-density particles such as gold, silver, iron, or even silica or polystyrene. Careful controls, investigating the ability to pellet the particles in a protein free environment should however always be carried out for a specific type of nanoparticle.

## 7. Summary

Based on both new experimental data and previously published results, we here draw three main conclusions: (i) low-speed centrifugation cannot be used to pellet ∼100 nm sized liposomes with no or low protein adsorption, (ii) centrifugation procedures based on the protocol used by Digiacomo *et al.* can only, in some instances, be used to isolate liposomes from plasma (*e.g.* the special case of nonPEGylated cationic liposomes), (iii) the ability for some (*e.g.* nonPEGylated cationic) liposomes to pellet following the centrifugation-based protocol used by Digiacomo *et al.* is most likely due to their propensity to aggregate in human plasma. We have furthermore discussed several methodological pitfalls of centrifugation. Taken together, we argue that centrifugation falls short of being a one-size-fits-all “best practice” solution to isolate liposomes. We hence come to the opposite conclusion than Digiacomo *et al.* namely that centrifugation should not be the method of choice for researchers studying the protein corona of liposomes for drug delivery. The alternative available methodologies such as AF4 and SEC are not perfect, but they are in our opinion better than the centrifugation method proposed by Digiacomo *et al.* Further, we would like to stress the need for proper control experiments when studying the protein corona on nanocarriers – a task that is very complex and associated with many challenges.

## Conflicts of interest

There are no conflicts to declare.

## Supplementary Material

NA-005-D2NA00343K-s001
