# Supplementary material for: Comment on “Optimal centrifugal isolating of liposome–protein complexes from human plasma” by L. Digiacomo, F. Giulimondi, A. L. Capriotti, S. Piovesana, C. M. Montone, R. Z. Chiozzi, A. Laganá, M. Mahmoudi, D. Pozzi and G. Caracciolo, Nanoscale Adv., 2021, 3, 3824
Source: Nanoscale Adv. 2022 Nov 30;5(1):290–9. doi: 10.1039/d2na00343k (PMC9765536; doi:10.1039/d2na00343k)
Supplement: NA-005-D2NA00343K-s001 [file NA-005-D2NA00343K-s001.pdf]

Electronic Supplementary Material (ESI) for Nanoscale Advances.
This journal is © The Royal Society of Chemistry 2022

Supplementary Information for the manuscript

Comment on "Optimal centrifugal isolating of liposome-protein complexes from human plasma" by L. Digiacomo, F. Giulimondi, A. L. Capriotti, S. Piovesana, C. M. Montone, R. Z. Chiozzi, A. Laganá, M. Mahmoudi, D. Pozzi and G. Caracciolo, Nanoscale Advances, 2021, 3, 3824.

Rasmus Münter & Jens B. Simonsen*

Supplementary Figure 1:

To investigate if the cationic liposomes used by us and Digiacomo *et al.* aggregated in plasma, we employed flow cytometry as previously described[1]. We took out 50 µL of the samples also used for the centrifugation study (immediately before the centrifugation step) and acquired a constant volume (30 µL) on an Accuri C6 flow cytometer (which according to the manufacturer has a lower size limit of 0.5 µm). Results are shown below. In pure plasma, an average of 30.000 events could be acquired. The nature of these events is unknown, but may be cell debris, large protein aggregates, large extracellular vesicles, chylomicrons and lipoproteins. These DiI-negative events were used to define the threshold for DiI-positive plasma particles. When PEGylated particles in PBS were run on the flow cytometer 200-300 events were acquired, whereof approx. two thirds were fluorescent (the rest may have been instrument noise). When the PEGylated liposomes were incubated in plasma, the number of events increased to approx. 22.000, corresponding to roughly 80% of the total events in the pure plasma sample as expected, whereof 4000-5000 where positive to the DiI fluorophore. Hence, the PEGylated liposomes did not give rise to any overall increase in large particles found in plasma, but there was a 20-fold increase in DiI positive events specifically, indicating some sort of liposome aggregation. When nonPEGylated liposomes were incubated in PBS, an average of 32.000 particles could be acquired on the flow cytometer, 99% of which were DiI-positive. In other words, this formulation was unstable even in a buffered environment. When the nonPEGylated particles were exposed to plasma, however, the number of large particles acquired increased even more. An average of 84.000 events were acquired, with one sample even containing 107.000 events. The majority of these (80-87%) were DiI-positive. The large increase in acquired events compared to liposome-free plasma, as well as the presence of the liposome label in the particles, strongly indicates that the nonPEGylated liposomes form aggregates in plasma. Not only the large increase in DiI positive events, but also the high fluorescence and light scatter intensities, indicate the presence of liposome-aggregates. The ability of this formulation to pellet may therefore simply be because it forms large

and dense aggregates in the presence of plasma proteins, which are easier to pellet than individual liposomes.

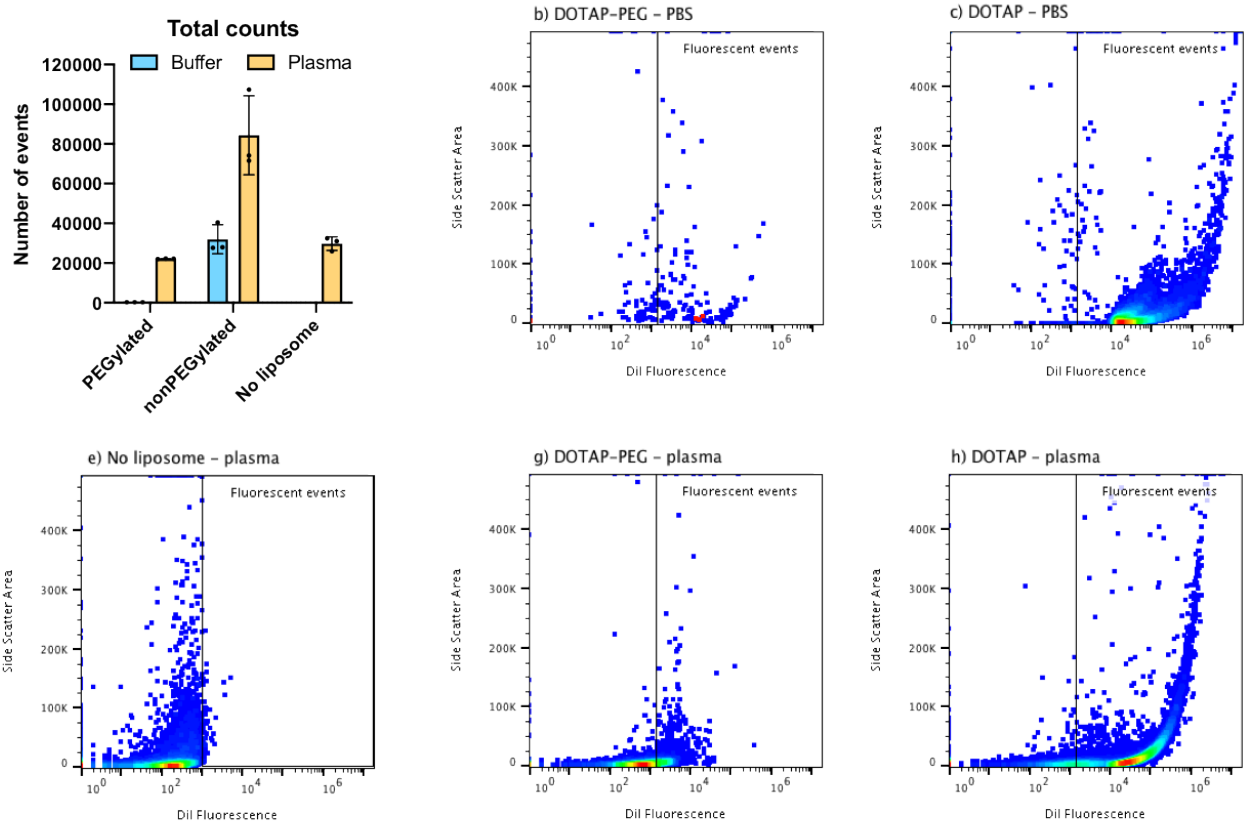

Presence of large fluorescent particles in plasma samples with and without liposomes detected using Flow Cytometry. A constant volume of plasma was acquired using an Accuri C6 flow cytometer, from samples containing either 80% plasma or PBS, after 1h incubation with PEGylated or nonPEGylated liposomes. a) overview of number of acquired events. B) PEGylated liposomes in PBS. C) nonPEGylated liposomes in PBS, e) plasma without liposomes. f) PEgylated liposomes in plasma. G) nonPEGylated liposomes in plasma.

Supplementary Figure 2:

To investigate if there was significant contamination of unbound proteins in liposome isolates, we measured the protein content in the pellets of samples with no liposomes as well as in samples with the cationic PEGylated used by Digiacomo et al. To check if any carry-over could be avoided by using Protein LoBind tubes, we performed the experiment using both such tubes as well as regular microcentrifuge tubes. Briefly, we incubated 80% plasma samples, either in presence or absence of the PEGylated liposomes, at 37 °C for 60 minutes in either regular Eppendorf microcentrifuge tubes, or Protein LoBind tubes. Then, the samples were exposed to the 60 min 18.000 g centrifugation protocol. In order to investigate the pelleted proteins instead of the liposomes, we then did as described in the methods section of the Digiacomo et al. paper: "To study potential effects of the isolation protocols on the resulting protein corona […] pellets were then washed three times with PBS to remove unbound and loosely bound proteins (the "so-called soft protein corona") and finally obtain the so-called "hard corona"." Finally, the pellet was also resuspended in PBS. For each of the three washing steps, as well as for the pellet, we quantified the protein concentration by measuring the Trp fluorescence, compared to an HSA standard. The figure below shows the recovered protein in each sample, with each data-point representing technical replicates.

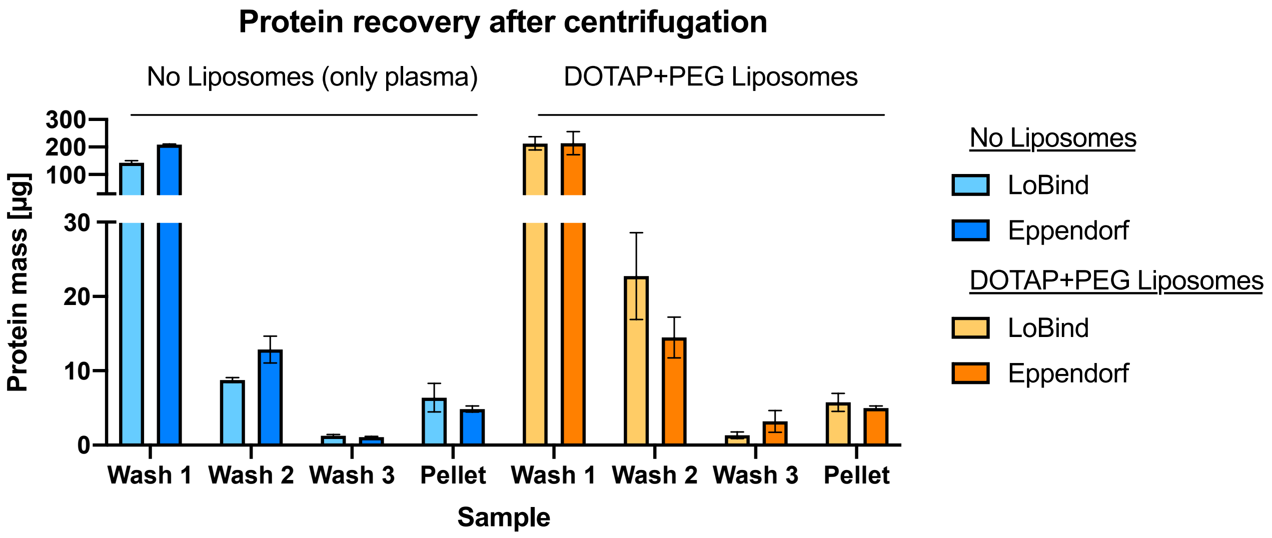

First, it is evident that the amount of pelleted protein is very low. In all washing steps and in the final pellet, two total protein content was < 200 µg protein, and in the final pellet the protein content was less than 10 µg. In comparison, the average protein content in the samples before centrifugation was approx. 8.4 mg protein. Next, it is clear that the majority of the protein pelleted by the centrifugation protocol was removed in the first washing step. In the absence of liposomes, the protein content in

both the first and second wash was lower for LoBind tubes than for regular Eppendorf tubes, indicating that the LoBind tubes indeed has less unspecific binding.

Importantly, the presence of the liposomes did not increase the mass of pelleted proteins. Specifically, when performing the experiment in LoBind tubes, the protein content in the pellet was 0.6 µg lower in presence of liposomes compared to in absence of liposomes, and only 0.2 µg higher when the experiment was performed in regular Eppendorf tubes. Analyzing the results using a two-way ANOVA with Tukeys post-hoc test, we found that the 0.2 µg increase in protein mass was not statistically significant. Even if this result had represented an actual increase in pelleted protein, 0.2 µg protein would correspond to only 13 proteins per liposome in the pellet (assuming 100.000 lipids per liposome and an average protein mass of 66.5 kDa), hence representing a very sparse protein corona. If taking into account that only 15% of the liposomes were pelleted, the average number of proteins per total liposomes in the entire sample, would only be two proteins per liposome. If also including the soft protein corona in the calculation (i.e. all proteins removed in the three washing steps), there would be 91 proteins per liposome, still corresponding to only 11% of the liposomal surface area being coated with proteins (assuming a 3.5 nm hydrodynamic radius of the proteins, corresponding to that of albumin) still representing a relatively sparse protein corona.

References:

[1] R. Münter, M. Bak, E. Christensen, P. J. Kempen, J. B. Larsen, K. Kristensen, L. Parhamifar, T. L. Andresen, *Acta Biomater.* **2022**, DOI 10.1016/j.actbio.2022.03.029.
